# Requirements for an Electronic Health Tool to Support the Process of Help Seeking by Caregivers of Functionally Impaired Older Adults: Co-Design Approach

**DOI:** 10.2196/12327

**Published:** 2019-06-07

**Authors:** Mélanie Tremblay, Karine Latulippe, Anik MC Giguere, Véronique Provencher, Valérie Poulin, Véronique Dubé, Manon Guay, Sophie Ethier, Andrée Sévigny, Maude Carignan, Dominique Giroux

**Affiliations:** 1 Department of Teaching and Learning Studies Université Laval Québec, QC Canada; 2 Department of Family Medicine and Emergency Medicine Université Laval Québec, QC Canada; 3 Centre of Excellence on Aging Quebec Québec, QC Canada; 4 School of Rehabilitation University of Sherbrooke Sherbrooke, QC Canada; 5 Centre of Research on Aging Centre intégré universitaire de santé et de services sociaux de l’Estrie-Centre hospitalier universitaire de Sherbrooke Sherbrooke, QC Canada; 6 Université du Québec in Trois-Rivières Trois-Rivières, QC Canada; 7 Centre for Interdisciplinary Research in Rehabilitation and Social Integration Québec, QC Canada; 8 Faculty of Nursing University of Montreal Montreal, QC Canada; 9 Research Centre of the University Hospital of Montreal Montreal, QC Canada; 10 School of Social Work and Criminology Université Laval Québec, QC Canada; 11 Department of Rehabilitation Université Laval Québec, QC Canada

**Keywords:** functionally-impaired elderly, caregivers, co-design, eHealth, telemedicine, help-seeking behavior

## Abstract

**Background:**

In Quebec, Canada, many public, community, and private organizations provide resources to caregivers of functionally impaired older adults. Nevertheless, these resources may be difficult for caregivers to find. A co-design study was conducted to address the gap between caregivers and access to resources. The purpose of this study was to support the process of help seeking by caregivers of functionally impaired older adults through electronic health (eHealth).

**Objective:**

The purpose of this study was to focus on the identification of functional and content requirements for an eHealth tool to support the help-seeking process of caregivers of functionally impaired older adults.

**Methods:**

This study uses a co-design process based on qualitative action research approach to develop an eHealth tool with health and social service professionals (HSSPs), community workers, and caregivers. The participants acted as co-designers in identifying requirements for the tool. A total of 4 design workshops and 1 advisory committee session were held in different locations in Quebec, Canada. Activities were videotaped and analyzed with a conceptual framework of user experience.

**Results:**

A total of 11 caregivers, 16 community workers, and 11 HSSPs participated in identifying the requirements for the eHealth tool. Several functional and content requirements were identified for each user need (19). Content requirements differed depending on the category of participant, corresponding to the concept of user segmentation in the design of information and communication technology. Nevertheless, there were disagreements among co-designers about specific functionalities, which included (1) functionalities related to the social Web, (2) functionalities related to the evaluation of resources for caregivers, and (3) functionalities related to the emerging technologies. Several co-design sessions were required to resolve disagreements.

**Conclusions:**

Co-designers (participants) were able to identify functional and content requirements for each of the previously identified needs; however, several discussions were required to achieve consensus. Decision making was influenced by identity, social context, and participants’ knowledge, and it is a challenge to reconcile the different perspectives. The findings stressed the importance of allowing more time to deal with the iterative aspect of the design activity, especially during the identification of requirements of an eHealth tool.

**International Registered Report Identifier (IRRID):**

RR2-10.2196/11634

## Introduction

### Background

Statistics show that almost 50% of Canadians will provide care to a family member or a friend during their lifetime; age-related needs are the most common problem requiring caregiving (28%) [1]. Quebec is the province with the lowest number of caregivers, at 25% of the population. Nevertheless, this number will potentially increase as the population ages. Demographic projections show that, in 2036, seniors could comprise between 23% and 25% of the population. This will result in a significant increase in seniors requiring the support of caregivers.

In Quebec, caregivers have access to many resources offered by health and social service professionals (HSSPs) and community organizations. These resources are considered to be a source of services or support for caregivers and include (1) programs involving renovation credits; (2) respite-type services, practical advice, and emotional support; and (3) strategies to reduce stress and symptoms associated with depression [2]. Nevertheless, literature confirms that caregivers have difficulty in accessing resources and that the existing services are underutilized [3].

### Electronic Health and Caregiving

According to a systematic meta-review, electronic health (eHealth) is a promising extension of the health care services currently available for caregivers, and evidence shows that interventions aimed at developing knowledge and providing information are efficient and effective [4]. eHealth, especially information and communication technology (ICT), can facilitate communication between caregivers and service providers [5]. The results specifically indicate an increased understanding of the illness [6]. Studies also confirm that eHealth can reduce caregivers’ depression and anxiety [7-9] as well as loneliness [9]. eHealth can allow caregivers to feel more confident about their caregiving skills [10]. It may bridge the gap between service providers and caregivers as it reduces distance obstacles, thus reaching underserved populations [11]. Nonetheless, caregivers’ needs are complex and vary depending on the diagnosis, changing caregiving roles, and family situations [12]. Therefore, for caregivers’ effective use of eHealth, the design of any eHealth tool targeting this population should involve a participatory approach [13-15].

### Co-Design of Information and Communication Technology

Co-design was first known as participatory design, and it refers to “the creativity of designers and people not trained in design working together in the design development process” [16]. It can be defined as a “process of collaborative design thinking: a process of joint inquiry and imagination in which diverse people jointly explore and define a problem and jointly develop and evaluate solutions” [17]. The central principle of co-design is the involvement of end users and stakeholders in the design process [18]. It is generally considered as the concept of user involvement (or participation) in software development and system success [19].

Numerous studies have demonstrated positive correlations between user involvement and system success [20]. A systematic review revealed that of the 87 studies that were analyzed, 59 reported that user participation contributed to the success of the system developed [21]. This field of research is a promising method to discover the appropriate interactions between technologies and quality of life, especially in the health field [22,23]. The co-design approach has led to cultural change among staff and patients in hospital environments, with older patients benefitting specifically [22]. In another case, the co-design approach led to a better sense of security and reduced stress for caregivers as it provided for increased awareness of each family member’s personal schedule [23]. Examples of a co-design approach with the aging population can also be found in the studies by Ventura and Talamo [18] and Ho et al [24]. Although there is a growing body of research that uses a co-design approach, the lack of co-design studies of the specific populations of caregivers and functionally impaired older adults indicates that more work in this area is required.

In the participatory design approach, specifically co-design, users engage with designers and researchers to find creative solutions to poorly defined problems [16]. A diversity of approaches exists in co-design [25]; end users can contribute at specific steps or each step of the design process. Although there are many models for the process of technology design, typical steps of user-centered design include (1) understanding the context, (2) understanding user requirement specifications, (3) creating prototypes, and (4) testing [26]. For the design of the tool, we decided to use knowledge and constructs from user experience (UX) design theory.

### Conceptual Framework

**Figure 1:**
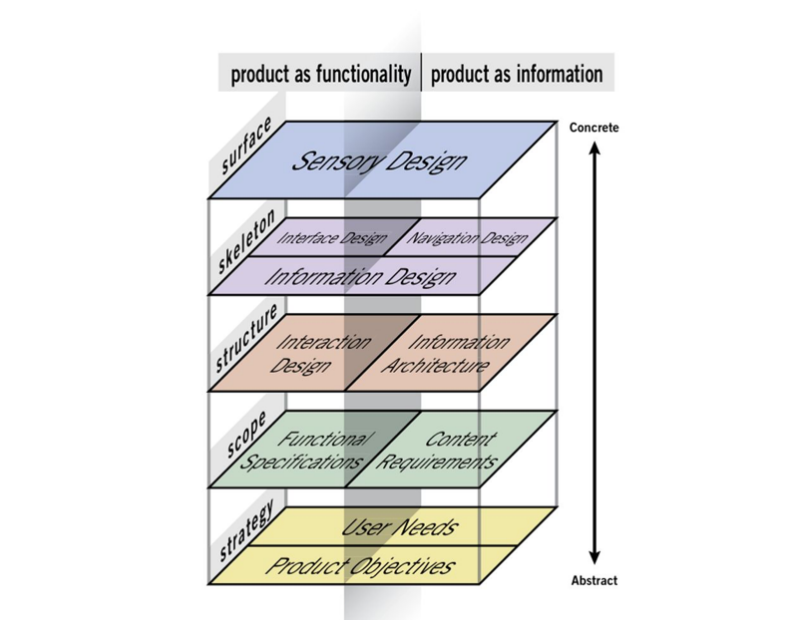
Elements of user experience.

To organize design sessions for the tool, we used the Conceptual Framework of User Experience proposed by Garrett [27]. The model (Figure 1) suggests a linear and iterative process for the design of Web-based technology. Garrett defines the different elements of UX in 5 dimensions: (1) strategy, (2) scope, (3) structure, (4) skeleton, and (5) surface. The elements are ordered with abstract-to-concrete considerations. Each dimension is considered in terms of the product as functionality on the one hand and the product as information on the other.

The scope step aims to identify the functionalities (functional specifications) and content required to meet the needs of users, based on the objectives of the product (strategy). Functional specifications (sometimes called *specs*) are the specific functionalities needed for the product and will guide the programmer’s decision regarding the coding language to use. Content requirements identify what type of content is needed (text, video, etc), the expected size, the person responsible for each element of the content, and the frequency of update needed. Ideas about requirements then need to be prioritized to determine what should be included in the product. Other studies have also used Garrett’s design constructs [28-30].

As part of a broad co-design study aiming to develop an eHealth tool to support the process of help seeking by caregivers of functionally impaired older adults, this paper reports on the scope dimension: the identification of content and functional requirements based on user needs. Traditionally, systems engineers write user requirement specifications; however, user input is crucial during this step. We must ensure that users are able to understand the specifications well enough to validate their accuracy [31]. According to El Emam and Madhavji [32], users should always participate in determining the requirements of system design, and different tactics can be used to promote participation. Mock-ups [33] and games [34] have shown impressive results. Notwithstanding the use of a UX conceptual framework, our approach (with user participation) involves a power-sharing creation model, whereas Sanders and Stappers [16] describe the research team as working in partnership with the participants.

## Methods

### Co-Design Strategy

**Figure 2:**
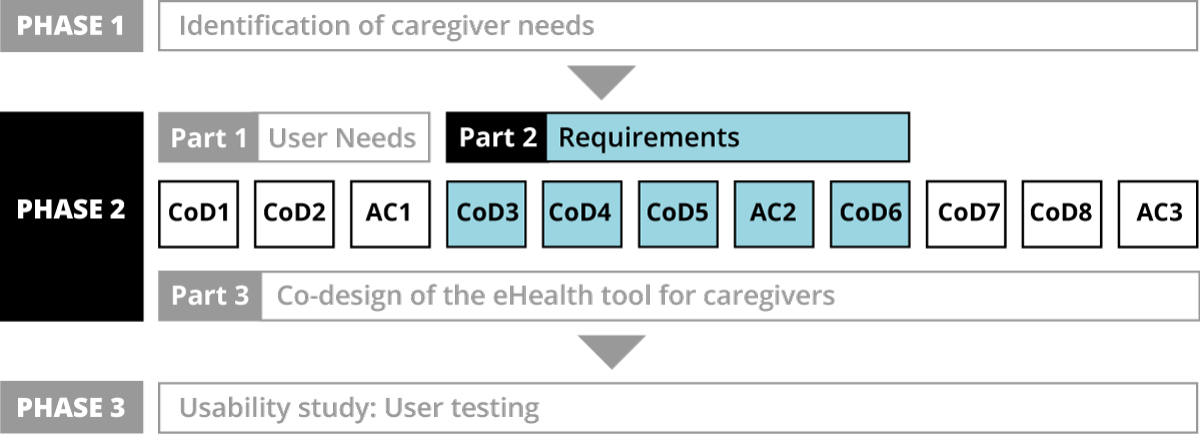
Segment of the study concerned in this paper.

This paper is part of a broad project conducted in 3 phases (Figure 2). The objective of phase 1 was to identify the needs of caregivers of functionally impaired older adults. The objective of phase 2 was to co-design an eHealth tool to support the help-seeking process of caregivers based on the results from this phase. The methodology and results of phase 2 are reported in the following 4 papers:

- The protocol of the global study [35]
- Part 1 focuses on the early stage of the design process: understanding the user needs [36].
- Part 2 focuses on the content and functional requirements based on user needs (this paper).
- Part 3 reports on the complete co-design process for the tool [37]

Finally, phase 3 is a usability study to verify the results obtained in the co-design process.

During phase 2, a total of 8 co-design sessions (CoDs) as well as 3 advisory committee sessions in 11 regions took place between May 2017 and June 2018.

The advisory committees guided the progression of the prototype, ensured continuity between CoDs, and made sure that the prototype conformed to the decisions made during the work sessions. The identification of requirements took place from CoD 3 to CoD 6 and during the second advisory committee session (AC 2; Figure 2).

### Participants (Co-Designers)

The participants recruited for this study were required to be potential users of an eHealth tool for caregivers of functionally impaired older adults in Quebec. Hence, we included 3 different categories of potential users: caregivers, workers from community settings, and HSSPs. For the purposes of this study, *caregivers* are defined as people who assist a functionally impaired older person on a sustained (weekly) basis. *Community workers* are defined as people from the community health network who offer services or interact directly with caregivers of functionally impaired older adults. *HSSPs* are defined as people from the public health care system who offer services or interact directly with caregivers of functionally impaired older adults. As part of their work, the latter categories of participants assist caregivers in their help-seeking process. They would be able to use the eHealth tool, which is designed for that purpose. For this study, they are considered to be potential end users.

Consistent with our methodological approach, the term co-designers (instead of participant) will be used to designate people who contributed to the identification of functional and content requirements.

We recruited co-designers from various regions of Quebec to meet the particularities of the people living in different regions. The sessions covered in this paper involved co-designers recruited from the following regions in Quebec: Saguenay-Lac-Saint-Jean (CoD 3), Bas-Saint-Laurent (CoD 4), Outaouais (CoD 5), and Montreal-Laval (CoD 6) for the CoDs and Capitale-Nationale and Chaudière-Appalache regions for the advisory committee sessions. Community workers were contacted directly (by phone or email). Direct contact was made with HSSP management of older adult services. The management used selection criteria to identify potential participants in their organization, and the HSSPs communicated with the research team. Caregivers received invitations to participate from either participating community organizations or the HSSPs. During the recruitment phase, the objective of the study was briefly explained to the co-designers: the design of an eHealth tool to support the help-seeking process for caregivers of functionally impaired older adults. All co-designers gave informed consent, and they received a nominal amount to cover travel and parking expenses. More details about the selection criteria and recruitment process are in the study protocol [35].

The co-design process also included the research team. Within the co-design spectrum, end users collaborate with designers and researchers to reach the design objective [16,18]. The research team interacts with participants during sessions and organized activities during the subsequent sessions. The research team included 3 researchers and 1 research assistant. The research director (DG) is a professor in occupational therapy, and the second researcher (KL) is a Doctor of Philosophy (PhD) student in educational technology and is specialized in occupational therapy. The other researcher (MT) is also a PhD student in educational technology and a specialist in the UX design field. The research assistant (MC) has an anthropology background. All members of the research team are currently working on other research projects with the aging population.

### Data Collection

The sessions involved different types of activities with specific objectives (Table 1). The research team had initially set out an objective for each session based on Garrett’s UX framework [35]. As the sessions progressed, it became apparent that a review of these objectives was required and, at times, iterative changes were made to address specific issues. Researchers initially planned 2 sessions to identify the requirements of the tool. However, they added activities during the AC 2, CoD5 and CoD6, to address the remaining aspects of the identification of the requirements of this eHealth tool.

A total of 5 meetings were needed to complete the requirements, although CoD 5 and CoD 6 were not entirely devoted to the identification of requirements.

The activities were selected according to the objective of the session, were chosen based from previous work and literature [18,32,33,38], and were based on expertise of each research team members (Table 1). Some activities involved the entire group of co-designers and others involved subgroup workshops with a moderator. Each session ran for 3 hours and was videotaped by 1 camera (Canon), 2 iPads (Apple), and an audiotape with 3 audio recorders (Olympus), thus, ensuring that all data coming from subgroups were recorded. To ensure accessibility to the sites, sessions were held in rented meeting rooms in a central city of the region visited.

During CoD 3, laptop computers and iPads were used by co-designers to compare existing eHealth ICT tools. Participants were shown a total of 6 websites (English and French), 2 apps, and 1 video. Researchers selected these tools to obtain a wide variety of functionality proposals. After a short review, co-designers were invited to identify the user needs met by each tool and rate how they were met (good or needing improvement). Open Broadcast Studio was used to collect data for the website review as this software enables simultaneous recording of the screen and co-designers’ reaction (with the webcam).

**Figure 3:**
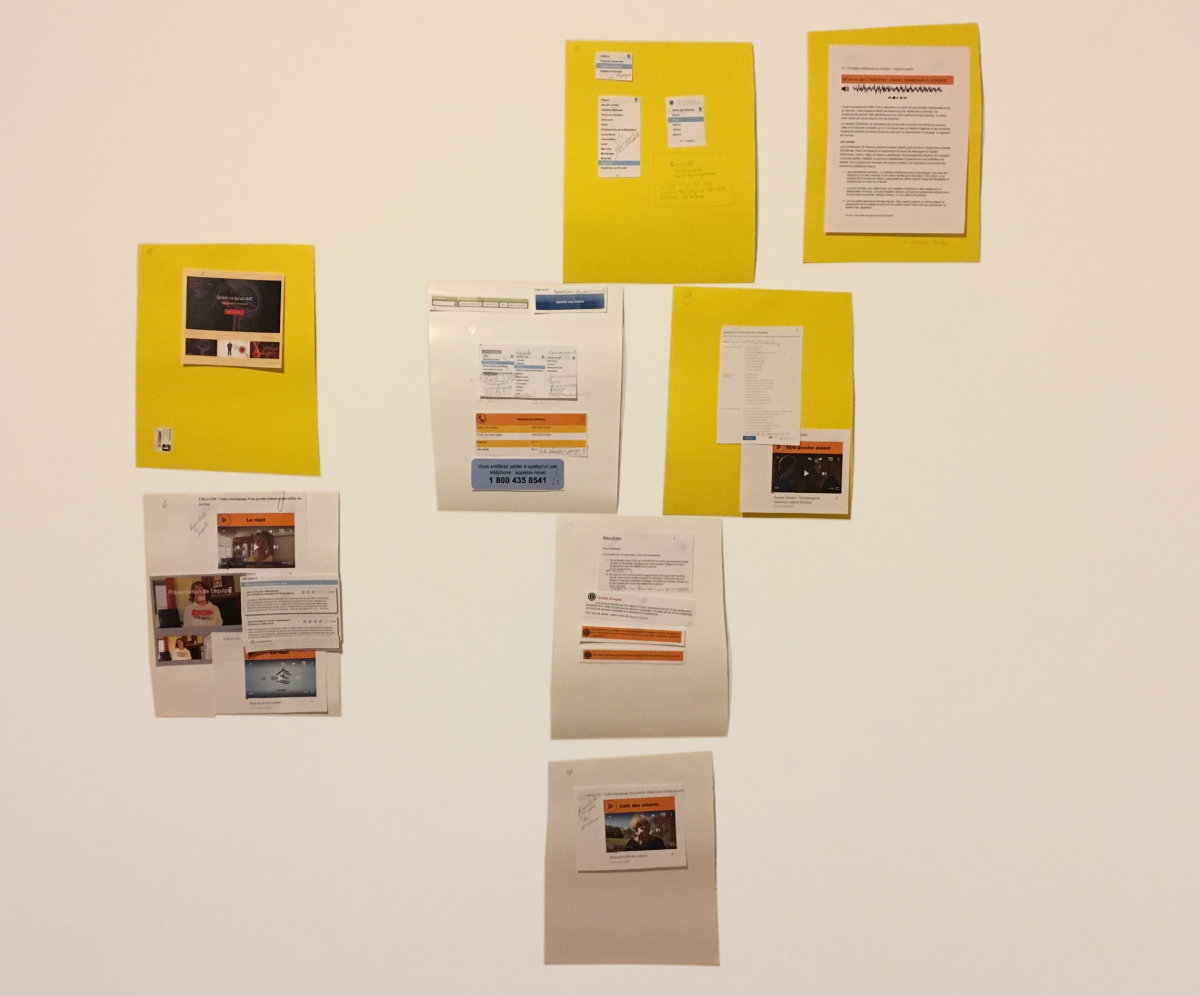
Paper prototyping.

During the paper prototype activity (CoD 5), the research team had prepared paper examples of functionalities and content requirements identified during CoDs 3 and 4. Participants had access to different sizes and colors of paper, scissors, pencils, and glue. They were asked to create paper website pages, decide on the functionalities and content for each page, and design how the pages were to be linked (Figure 3).

**Figure 4:**
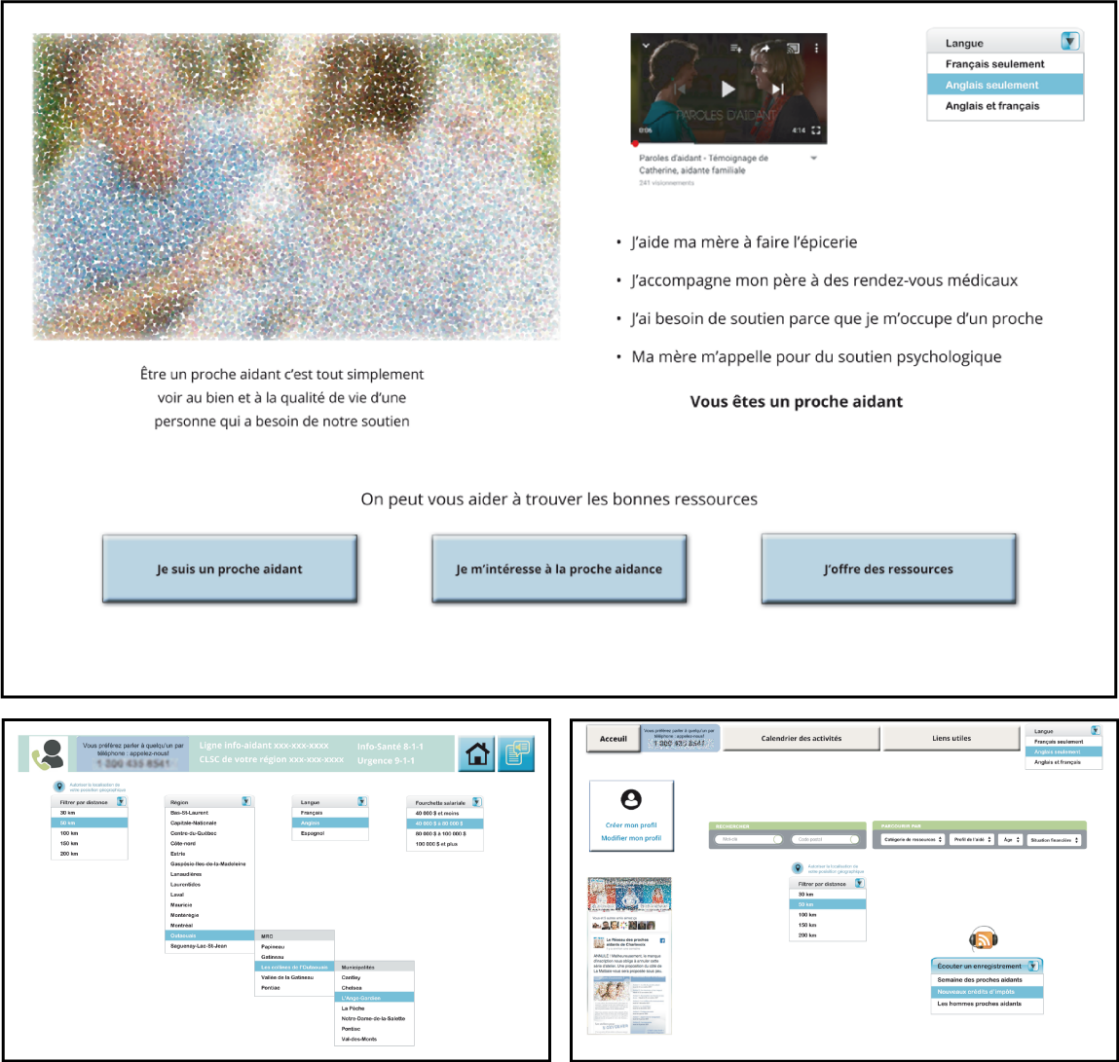
Interactive PDF (low-fidelity prototypes).

For the AC 2, paper prototypes were used to produce 3 interactive PDFs. Researchers presented these low-fidelity prototypes to the advisory committee participants as evidence of the progression of the work (Figure 4).

Data collected during sessions include audio and video recordings of co-design and advisory committee sessions, audio recording of preparation and after-action meetings, artefacts, paper documents used during sessions, and spreadsheets used by the research teams.

**Table 1:** Activities, objectives, and modalities for each session.

Session	Activity	Objectives	Modalities
CoD^a^3	Comparison of existing electronic health information and communication technology tools (websites and apps)	(1) Identification of the user needs that are already met by other tools and (2) identification of functionalities and content of existing tools related to those needs (what co-designers would keep, modify, or change).	Subgroup workshops
CoD 4	Brainstorming	(1) Identification of functional or content requirements for the needs not met by existing tools.	Group and subgroup workshops
CoD 5	Paper prototypes	(1) Prioritization of functional requirements and (2) structuring of content and design of information architecture.	Subgroup workshops
Second advisory committee session	Presentation of 3 prototypes and discussion	(1) Prioritization of functional requirements.	Group
CoD 6	Brainstorming	(1) Creation of content requirements and design of information.	Subgroup workshops

^a^CoD: co-design session.

### Data Analysis

For data analysis, we followed an analytic questioning method [39]. This method involves 3 major steps: (1) articulate operationalizing questions according to the research objective, (2) submit a relevant corpus to these questions to obtain a first draft of answers that will be used to generate more precise questions, and (3) answer the questions generated with direct answers (statements, observations, and propositions) or new questions if appropriate. In this case, the objectives of each session were articulated in a question form as a first step.

The principal objective of the scope dimension of Garrett’s UX framework is to identify functional and content requirements based on user needs. Therefore, in the data analysis, we had to ensure that the requirements address each user need. After each session, the research team conducted a debriefing to underline significant results. We first analyzed data that were collected in response to the operationalizing questions (Table 2). The results were then recorded in a Microsoft Word or Excel document. When there were subgroup workshops, each member of the research team reported the results of the workshop where they acted as a moderator. In such cases, workshop results were then combined into a single document (Microsoft Word and Excel). Following the production of the reports, the research team met to review documents and to (1) confirm the validity of the interpretation of the information collected, (2) assess the degree to which the session’s objectives were attained, and (3) design more precise questions to generate specific answers. It was sometimes necessary to refer to the audio and video recordings of the sessions to retrieve the negotiation of design decisions among co-designers. Often, more than 1 meeting (3-4 hours) was needed to get a complete picture of the results achieved and ensure their accuracy. Data were gathered in an Excel spreadsheet linking requirements and user needs.

**Table 2:** Data analysis.

Session	Operationalizing questions	Data analysis
CoD^a^3	Which needs from the user need list are addressed by the existing eHealth^b^ tools? What are the requirements of existing tools addressing these needs? What changes would be required to better address the needs? Which needs are not addressed by existing tools?	(1) List of requirements related to user needs and (2) list of user needs not addressed by existing eHealth tool (N=8)
CoD 4	What are the potential content and functional requirements that could address the remaining needs?	List of requirement ideas to complete the list of requirements and address each user need
CoD 5	From all the requirements obtained in CoD 3 and CoD 4, which requirements should be prioritized? Based on a selection of requirements, what would be the appropriate information architecture?	Information architecture propositions (N=3) including prioritized requirements
Second advisory committee session	Based on the 3 architecture propositions, how must we prioritize conflicting functional requirements?	Decisions about conflicting functional requirements
CoD 6	What information should be included based on content requirements? How should the information be presented and formulated?	Prioritization of content requirements and content creation for functional requirements

^a^CoD: co-design session.

^b^eHealth: electronic Health.

Moreover, while conducting the analysis of the sessions, it became obvious to the research team that data differed depending on the category of users: (1) caregivers or (2) service providers. This resulted in the need for data to be separated into these 2 categories (segments of users).

Co-design can be considered as a type of action research, as a form of knowledge production (or cocreated) through an iterative process linking action and research [40]. Co-design shares many values and goals with action research, such as empowerment and democratization, and its rigor stems from trustworthiness [41] composed of 4 distinct properties: credibility, transferability, dependability, and confirmability [42]. Credibility was obtained with the review of documents to confirm the validity of the interpretation of the information. Transferability (or applicability) was obtained by visiting different regions of the province of Quebec, minimizing situational variations to the findings. Dependability was obtained with the Excel spreadsheet allowing trackable variance of the data from sessions. Finally, confirmability was obtained with the advisory committee editing the decision points throughout the process.

The study received ethical approval from the *Comité d'éthique de la recherche sectoriel santé des populations et première ligne* (2016-2017-10 MP).

## Results

### Participant Characteristics

A total of 11 caregivers, 16 community workers, and 11 HSSPs participated in the identification of requirements for the ICT eHealth tool (Table 3). Participants were executive officers (n=4), retired individuals (n=4), stakeholders (n=2), coordinators (n=2), nurses (n=2), public servants (n=2), mediators (n=1), homemakers (n=1), and researchers (n=1). This researcher participated as a caregiver.

**Table 3:** Sociodemographic data of co-designers.

Sociodemographic items		Caregivers	Community workers	Health and social service professionals
**Gender, n**				
	Women	11	10	11
	Men	0	6	0
**Age (years)**	
	Range	44-82	25-62	28-49
**Education level, n**				
	High school	4	0	2
	College	3	1	4
	University	4	15	5

### Requirements Identified for User Needs

Functional and content requirements were identified for each user need (19 identified user needs; [35]). Most user needs were met by functional, content, or both categories of requirements. For example, only 1 content requirement was conserved for the need “Ask a question.” Content requirements are also sometimes directly related to functional requirements. This was the case with the user profile: it needed to be created or modified (functional), but we also needed to decide what was in it (content). Some requirements were identified for more than 1 need. Indeed, videos were a functional requirement (embedded video functionality) and a content requirement (the video itself) identified to meet the needs: “be reassured about resources,” “recognize themselves as caregivers,” and “be encouraged to use the services.” In those cases, requirements were repeated as this allows the research team to track the needs that were met and to get a sense of the importance of each requirement.

Table 4 presents the final functional and content requirements for each need. Where functional and content requirements are related, they are presented next to each other. Sometimes the requirements that were suggested in the first sessions were then rejected during following session.

### Nonretained Requirements

There were important differences between initial requirements identified during the third and fourth co-design activities and the final requirements. Almost half of the initially identified requirements during the brainstorming sessions (CoD 4) and with the paper prototypes (CoD 5) were not retained. It is important to note that when content and functional requirements were related, both were automatically rejected, thus increasing the number of nonretained requirements. For instance, “Embedded video functionality” was rejected when related to “Web documentary.” However, the “Embedded video functionality” requirement was kept as it was also related to many other retained content requirements, as shown in Table 4. There were other requirements rejected as they were not related to design of an ICT technology but more to the nature of a service, that is, “Inducement from service providers” (invitation to their events).

There were disagreements among co-designers about specific functionalities: (1) functionalities related to the social Web, (2) functionalities related to the evaluation of resources for caregivers, and (3) functionalities related to emerging technologies. Although the functionalities related to the social Web were to meet significant caregivers’ needs such as “Be encouraged to use the services,” “Be encouraged to ask for help before reaching a state of exhaustion,” and “Be able to connect with people experiencing the same situation,” co-designers were concerned about advantages and safety issues for the community of users. Some HSSPs and community workers were especially concerned about the potential for caregivers to get misleading advice and receive discouraging comments from other caregivers. These participants even mentioned the risk of malicious people taking advantage of the situation as caregivers can become vulnerable at one point. HSSPs and community workers were concerned about the security of the information. Participants also mentioned there was already a social media tool connecting caregivers in Canada.

Debates over functionalities related to the evaluation of resources for caregivers as well as the social Web took place during AC 2 and the sixth co-design activity. Many co-designers, especially community workers, were uneasy with respect to the idea of evaluating resources. Another example of which is meeting caregivers’ needs to “Be comfortable using the services” and “Be reassured about resources”; co-designers were worried about the possibility of misevaluations and the effect that a negative evaluation could have on service providers. Community organizations could be significantly affected by a negative evaluation as they rely on the financial support of the public. Moreover, co-designers reported concerns that caregivers would evaluate the person who provided the service and not the service itself. Therefore, “The assessing and ranking system (stars and vote),” “The Voting system (have you found this useful?),” “Suggestions for improvements,” “Choice of comments to choose from,” and “Add comments” were all rejected. Functionalities related to emerging technologies such as “Bots on Messenger,” “Creation of a database to document the needs in relation to regions,” and “Use of Big Data” were considered to be interesting but nonessential at this point. Participants suggested they could be retained for a second phase of development.

**Table 4.** Final functional and content requirements for user needs.

Needs and functional requirements		Content requirements
**Ask questions**	
	—^a^	Phone numbers for the help line for caregivers
**Be able to add training workshops, resources, and activities**		
	Add activities	Form to add activities
	Add resources	Form to add resources
**Be able to connect with people experiencing the same situation**		
	No requirements retained^b^	No requirements retained^b^
**Be able to keep and retrieve information easily**		
	Add to favorites	My favorite page
**Be comfortable using the services**		
	Enter preferences (ie, gender of the care assistant)	List of possible preferences
	Embedded video functionality	Caregivers and users of resource testimonials
	Twinning of caregivers	—
	Embedded video functionality	Service provider testimonials explaining their resources
	—	Details about services and resources
**Be encouraged to ask for help before reaching a state of exhaustion**		
	No requirements retained^b^	No requirements retained^b^
**Be encouraged to use the services**		
	Embedded video functionality	Virtual visits (presentation of the team, list of services)
	Embedded video functionality	Video testimonial
	—	Description of services: here are 5 places for respite services in your region, here you can visit, here are the services they offer, and here is the cost
**Be guided in the help-seeking process**		
	Region repertory filter	Region repertory
	Construction of the profile with questions	Resources repertory
	—	Phone numbers for the help line for caregivers
**Be reassured about resources**		
	Embedded video functionality	Video of a worker explaining the services
	Embedded video functionality	User of resources testimonial
	—	Details about services and resources
	—	Explanation of how the resource meets the needs, even if indirectly.
**Have a choice of language**		
	Language filter (one or more languages)	English and French version of the tool
	—	English and French version of the tool
**Have access to a keyword search**		
	Search engine (by keywords and postal codes)	Keyword list (suggestions)
**Have access to services corresponding to the** **functionally impaired older person**		
	User profile creation/modification	Content of the user profile page
	Information filter	Parameters of the filters
**Have access to concise and simple tools**		
	Limit to only essential functionalities (avoid cognitive load)	Limit to only essential information (avoid cognitive load)
	—	Use of simple, intelligible terms, accessible to different literacy levels
**Have access to educational interventions**		
	No requirements retained^b^	No requirements retained^b^
**Have access to up-to-date information, anytime, anywhere**		
	News feed	New publications
	Add to favorites	Favorites page
	Search engine for old publications	—
**Know the service offer (costs, transport, home-based care, eligibility criteria, and proximity**		
	Editable profile to be filled by caregivers (ie, Zarit Scale, outil d'évaluation multiclientèle (OEMC), line 199 in the income taxes report)	Questions (ie, Zarit Scale, OEMC), line 199 in the income taxes report)
	Access to resources with an algorithm	Algorithm rules and sequence of operations (the algorithm should specify its limits)
	Form to add resources	Required information for resources in the form: cost (free or paid service), transport (or not), home-based care (or not), and eligibility criteria (list)
	Region filter	Reliability of sources
	Search by multiple criteria: keyword, age, financial situation)	—
	Networking among service providers and caregivers	—
	Geo-tracking	—
	Central access point	—
**Receive information regularly**		
	No requirements retained^b^	No requirements retained^b^
**Recognize the needs**		
	Clickable list of needs	List of needs
**Recognize themselves as caregivers**		
	Embedded video functionality	Video of caregivers
	Assistant (algorithm) determining the needs	—
**Requirements not directly related to a specific user need**		
	Add event to a calendar	Description of the event
	Personal calendar	Personal calendar
	Two profiles of users: caregivers and service providers	—
	Audio description for visually impaired people	—

^a^No corresponding requirement was identified (functional or content).

^b^See explanations in the following section: *Impression of Unmet User Need*.

### Requirements for Each Category of Users

As mentioned in the *Methods* section, it became obvious at one point that data regarding requirements were different depending on the user category (caregiver and service providers). Requirements identified for the caregiver category include (1) profile information requested, such as first and last name, email, password, region and sector, phone number, (2) consent to being notified when activities are offered in his or her region, (3) a personal calendar, and (4) the option of adding specific results to a Favorite page. The functional requirements for service providers are (1) a complex profile creation and (2) the option for adding activities and documents. This means that content requirements for each functionality must be related to an option (or word) in the search engine. For instance, when creating their profile, service providers must specify the services they offer and the customers’ profile. As they are searching for resources, caregivers can specify the profile of the older person they assist. Furthermore, co-designers were especially concerned about the word choice. Service providers and caregivers do not always use the same term when referring to a profile or service. Therefore, the content requirement “Keyword list (suggestions)” had to be associated with the content requirements in the service provider profile “Add resources” to avoid a “no result found” message from the search engine.

### Impression of Unmet User Needs

When requirements were first suggested and then not retained, the research teams tried to ensure that requirements for all the needs remained. Indeed, in some cases, requirements that had been rejected by co-designers left an impression of unmet user needs. Those needs are (1) having access to educational interventions, (2) receiving information regularly, (3) being able to connect with people experiencing the same situation, and (4) being encouraged to ask for help before reaching a state of exhaustion. Further analysis revealed that those needs had been met by requirements identified for other needs: “having access to educational interventions” and “being able to connect with people experiencing the same situation” were met by “Adding resources,” as resources could be an educational intervention or coffee break activities. “Receiving information regularly” was met by “Newsfeed,” and “being encouraged to ask for help before reaching a state of exhaustion” was met by “Assistant (algorithm) determining the needs” and testimonial videos of use of a service.

## Discussion

### Principal Findings

The participation of end users (or future users) during the specifications of requirements for eHealth tools is essential to ensure they are able to understand the requirements and confirm their correspondence with their needs [43,44]. Our results attest to the potential of their participation during this phase of the design process. The major findings of our study are the importance of (1) the iterative process of specifications of requirements for an eHealth tool and (2) the importance of user segmentation identification early in the process. Indeed, the diversity of potential users in this study (caregivers, HSSPs, and community workers) acting as co-designers resulted in a great diversity of views about requirements for the product. Merging co-designers’ perspectives was a major challenge and is also consistent with another study in which the requirements for an eHealth tool were developed with a co-design approach [45].

### Iterative Aspect of the Process

Our results reveal major differences between the beginning of the identification of requirements and the final decisions. Work had begun in the direction of the decisions that had been made initially, only to eventually be reversed. Iteration is indeed a characteristic of design activities [46]. The iterative process of design decisions in this case is also consistent with other works using participatory approaches, such as co-design in the medical and health domains [15,47]. In our study, most iterations were incited by strong disagreements among co-designers about specific functional requirements. It was necessary to come back to these functionalities 2 or 3 times in different sessions to arrive at a consensus that made it possible to meet the respective needs.

### Concerns About Functionalities Related to the Social Web

One problematic category of functionalities was functionalities related to the social Web. These are the ones commonly found in the Web 2.0. They are functionalities that allow users to communicate among themselves, thus creating a sense of community. Social networks have the potential to provide support and prevent feelings of loneliness [48], demonstrate benefits [49], and help caregivers deal with caregiving roles and responsibilities [50]. Even if evidence of the effectiveness of Health 2.0 technologies exists, our results indicate that there are still concerns about these technologies. Some caregivers mentioned that they did not have time to spend on social media as their role as caregivers was already time consuming. The safety and hazard concerns identified by HSSPs and community workers that may be misrepresented have also been discussed by Chou et al [51]. However, we question this perspective, which implies that the caregiver is a vulnerable person or is not able to judge the quality of the information provided. Would the benefits to be shared and encouraged by peers outweigh the perceived disadvantages? Is it possible to develop a system that would support both the quality of information and access to peer-to-peer exchanges? We decided to put this aspect aside considering the time and monetary constraints of the project, without eliminating it completely. This reflection will certainly be the subject of a project to further develop the tool.

Identification of requirements is a major step in the design process of ICT. It will define other steps, such as interaction design. Interaction design is part of the following design process structure according to Garrett’s framework. Interaction design involves the user behaviors and how the system will respond to this behavior. The choice of whether or not to include requirements related to eHealth 2.0 will impact how users interact with the product and how the system will respond. If, for example, co-designers had kept functional requirements such as “Live chat” and “Messaging between users,” we could expect that their interaction with the product would be more frequent and active. As of now, the tool interaction is mainly a search action. If caregivers were the only participants in the co-design of the tool, decisions could have been different. We asked the project’s coresearchers to explore the possibility of giving more weight to the caregivers acting as co-designers to include a social justice perspective. Given that the health and community stakeholders made serious arguments regarding the stakes involved, the researchers decided to find a consensus regarding the functionalities. This decision was also consistent with an implementation perspective. It is likely that community services and professionals will be recommending the tools to caregivers. Thus, if these groups did not accept the specific functionalities, they might block the implementation of the eHealth tool.

### User Identity and User Segmentation

The profile requirement also raised many issues. Service providers and caregivers will not need to save the same type of data within the tool. These different categories of users correspond to the concept of user segmentation in the design of ICT [27]. Content requirements differed depending on the category of participant (segment of users). This means that the website must have a specific secure section for service providers and a specific secure section for caregivers. Some co-designers in the CoD 5 session suggested other types of user segments: one for relatives and another for the functionally impaired older adults themselves, allowing each to have a different profile on the website. As this would have been problematic in terms of the co-design approach, these profile suggestions were rejected. These user categories were not involved in the first 5 CoDs and 2 advisory committee sessions, thus they could not participate as co-designers. Moreover, there was considerable concern about the idea that the caregiver would need to be connected to a user’s profile. Participants reported concerns about low–digital literacy users. They mentioned that older caregivers could be discouraged by having to create a user profile to gain access to the resource search engine. Therefore, it was decided that the user profile should be an option for caregivers and that the search engine should be accessible for unregistered users, thus adding a new user segmentation: the nonconnected (unregistered) user. User segmentation is an important design construct that must be considered early in the process. We found that our results demonstrate this and so are consistent with the results by Siek et al [15]. In our study, the requirements differed significantly for the 3 user segments (unconnected caregivers, connected caregivers, and service providers), resulting in difficulties in the discussions and negotiations regarding the requirements. The identification of user segments early in the process can facilitate the knowledge production regarding the specific requirements for each user segment.

The knowledge, social context, and identity of end users influence their design decision making. Future users may not be familiar with the design activity, and technical details of requirement specifications may be missing, thus increasing the time needed to complete the phase. Moreover, caregivers of functionally impaired older adults ranged in age from 44 to 82 years, which meant that some older adults themselves. Research shows that there is a second level of digital divide (skills and use of technology) related to age [52]: older people tend to have less digital literacy skills [53]. When acting as co-designers for the design of eHealth technology, older adults might have difficulty in understanding issues and implications related to the requirements identification of the technology being designed.

### Limitations

To address the validity of our results and ensure they are transferable and applicable in other regions, we decided to sample in different regions of Quebec. Our assumptions were that we would address situational variations of user needs by visiting different regions. Notwithstanding its value in our methodology, this sampling process has certain limitations. As CoDs were held in different regions, participants were acting as co-designers during 1 session only. This resulted in participants working on content and functionality requirements that were based on the user need decisions of other participants. This could have influenced their motivation and engagement during the session, as they were not participating in the entire design process of the eHealth tool. Participant impressions about their participation can affect their motivation and have an impact on results [19,54]. To limit this impact, the research team worked to explain clearly why these choices were made and to highlight the importance of everyone’s input in this product creation. To enhance co-designers’ understanding of the results presented to them, we added a presentation to each session with more detailed explanations of previous decisions.

### Conclusions

There is a growing body of research using a co-design approach for the design of eHealth technologies; however, most studies focus on the product developed and less on the design process. Moreover, there is a lack of common language to discuss the findings in the design of eHealth. This paper addresses these issues by using design theory in the discussion of the co-design of an eHealth ICT tool to assist caregivers of functionally impaired older adults in their help-seeking process. Results are discussed with design constructs such as the iterative nature of the process and user segmentation.

In this study, the iterative aspect appears to be even more important because the future users of the tool acted as co-designers. The number of iterations required increased, as it was sometimes a challenge to reconcile the different perspectives of co-designers coming from different regions of Quebec. Our findings stressed the importance of (1) allowing more time to deal with the iterative aspect of the design activity, especially during the identification of requirements and (2) identifying potential user segments early in the process, as user segmentation has implications on remaining design decisions. More research should be conducted to address the relationship between older people’s digital literacy and their participation in co-design of eHealth for this population.

A usability study will be conducted next year. Usability refers to the “functional relationships between people and the products and systems they use” [55]. This usability study will help determine whether the product meets the usability criteria: usefulness, efficiency, effectiveness, satisfaction, and accessibility [56]. It will also contribute to the documentation regarding the potential for including users in the design process.

## Abbreviations

AC 2: second advisory committee session
CoD: co-design session
eHealth: electronic health
HSSP: health and social service professional
ICT: information and communication technology
PhD: Doctor of Philosophy
UX: user experience
